# Evaluation of the circulating level of fibroblast activation protein α for diagnosis of esophageal squamous cell carcinoma

**DOI:** 10.18632/oncotarget.16274

**Published:** 2017-03-16

**Authors:** Yuehua Liao, Shan Xing, Banglao Xu, Wanli Liu, Ge Zhang

**Affiliations:** ^1^ Department of Microbial and Biochemical Pharmacy, School of Pharmaceutical Sciences, Sun Yat-Sen University, University Town, Guangzhou, China; ^2^ Department of Clinical Laboratory Medicine, Sun Yat-Sen University Cancer Center, Guangzhou, China; ^3^ Department of Clinical Laboratory Medicine, Guangzhou First Municipal People’s Hospital, Guangzhou Medical University, Guangzhou, China

**Keywords:** FAPα, esophageal squamous cell carcinoma, plasma, diagnosis biomarker, ELISA

## Abstract

To evaluate whether circulating fibroblast activation protein α (FAPα) could serve as a biomarker for the diagnosis of esophageal squamous cell carcinoma (ESCC), enzyme-linked immunosorbent assay (ELISA) was used to detect plasma FAPα in 556 participants including ESCC group, benign esophageal disease group, healthy controls and other cancer controls group. The levels of plasma FAPα were significantly decreased in ESCC patients (*P* < 0.001) and showed a positive correlation with HDL-C levels (R = 0.372, *P* < 0.001). The sensitivity and specificity of plasma FAPα were 56.1% and 85.6% based on the optimal cut-off (49.04 ng/ml, AUC = 0.714). The combination of FAPα and the traditional biomarkers (CEA, CYFR211 and SCCA) improved the sensitivity (41.5%) without compromising the specificity (95.0%). Contradictorily, the immunohistochemical staining revealed the overexpression of FAPα in stroma of ESCC tissues. So the source of soluble FAPα was further explored by qRT-PCR, Western blotting, ELISA and immunoprecipitation in fibroblast cell lines and mouse xenograft models. We found that the plasma FAPα was not correlated with the FAPα expressed in tumor, and the multi-organ might contribute to the circulating levels of FAPα including skeletal muscle, liver and bone marrow. These results indicated that the low plasma FAPα level might due to the systemic reaction to the presence of tumor and circulating FAPα level might be a potential indicator for diagnosing ESCC.

## INTRODUCTION

Esophageal squamous cell carcinoma (ESCC) is one of the most aggressive gastrointestinal cancers in China [1]. It has a very high mortality rate due to the relatively late stage of diagnosis [2]. Currently, traditional tumor markers, such as CEA, CYFRA211 and SCCA, are used to diagnose and evaluate ESCC progression. However, these tumor markers exhibit a low sensitivity in detecting ESCC. Therefore, there is an urgent needed to explore valuable tumor markers for ESCC detection [3].

Recently, circulating fibroblast activation protein α (FAPα) shows good specificity for colorectal cancer (CRC) diagnosis, and the combination of FAPα and other multiple markers all show high sensitivity for early detection of CRC [4–5]. Circulating FAPα levels were demonstrated significantly lower in cancer patients compared with healthy subjects and correlated inversely with survival in most types of cancer [6–9]. However, the circulating FAPα level in ESCC is still unclear.

FAPα, also known as seprase, has long been known to be expressed in the cancer associated fibroblasts of tumor stroma, and plays multiple roles in neoangiogenesis, invasion, and metastasis [10–12]; FAPα has been investigated as an invasion biomarker in various cancers, and has also been explored as a target for cancer therapy [13–14]. Multiple studies have confirmed that the expression of FAPα was upregulated in ESCC tissues [15]. Contradictorily, FAPα^+^ stromal cells are found to be present in the multiple normal tissues and organs [16–17]. Besides, soluble form of FAPα in human blood (also named antiplasmin-cleaving enzyme, APCE) was reported to play a role in fibrinolysis [18]. Until now, little is known about the exact origin of circulating FAPα.

In the present study, we measured the plasma FAPα level in cancer patients and investigated the associations between circulating FAPα and patients’ clinical outcomes to evaluate the clinical value of plasma FAPα as diagnostic parameter in ESCC patients.

## RESULTS

### Plasma FAPα level was reduced in of ESCC patients

**Figure 1:**
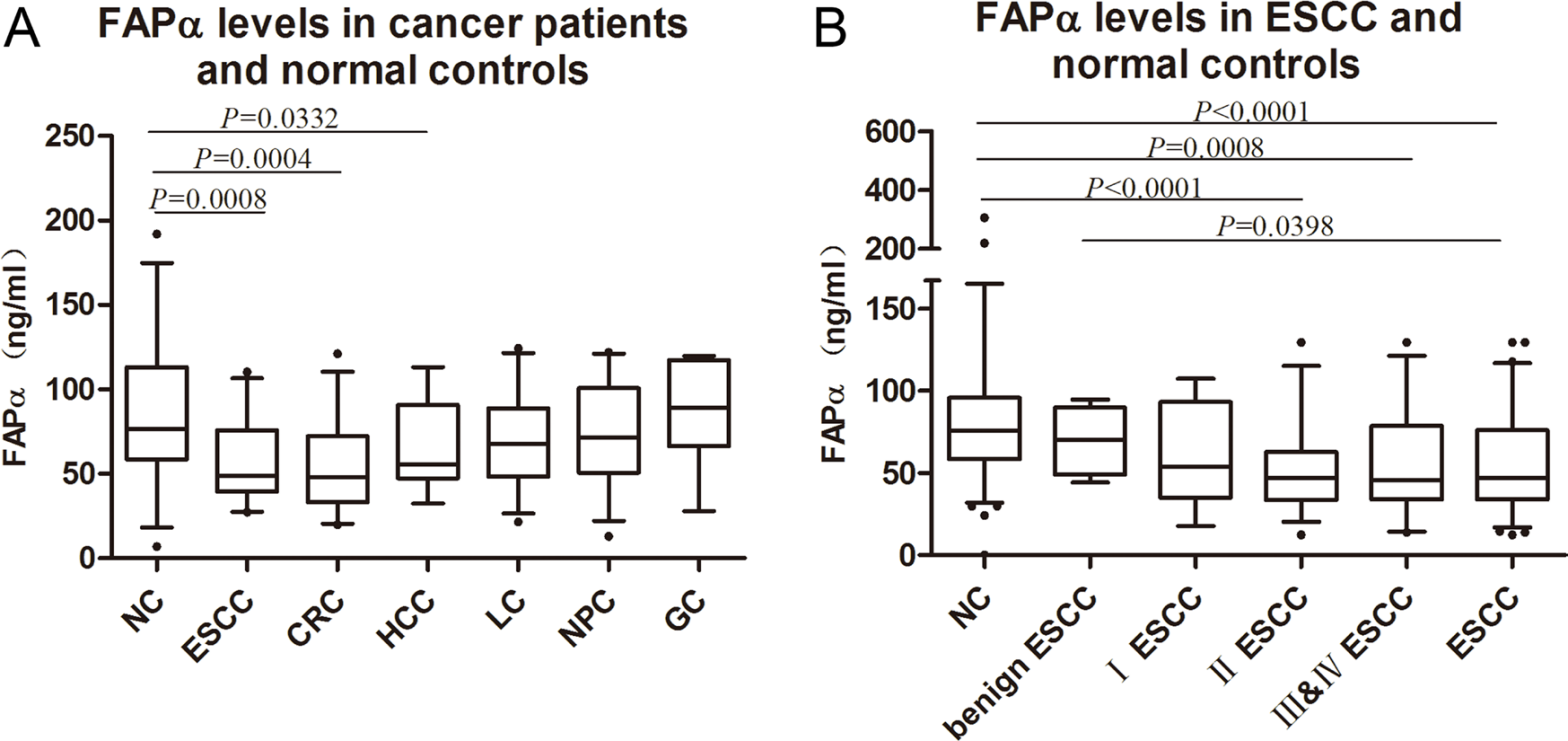
FAPα levels detected in the plasma of ESCC patients (**A**) Significant decline were found in the comparison between normal controls (87.78, 95% Cl: 73.40–102.2) and ESCC (57.55, 95% Cl: 49.72–65.38), HCC (64.56, 95% Cl: 52.86–76.27), CRC(52.09, 95% Cl: 41.92–62.25) patients. (**B**) Plasma FAPα comparison between healthy controls (80.67, 95% Cl: 72.49–88.86), benign ESCC (70.01, 95% Cl: 55.74–84.28), I ESCC (61.45, 95% Cl: 44.31–78.59), II ESCC (51.80, 95% Cl: 42.51–61.09), III & IV ESCC (55.56, 95% Cl: 44.22–66.90) and ESCC (56.79, 95% Cl: 49.59–64.00). “NC” is short for “normal control”. “CRC” is short for “colorectal cancer”. “LC” is short for “lung cancer”. “NPC” is short for “nasopharyngeal carcinoma”. “HCC” is short for “hepatocellular carcinoma”. “GC” is short for “gastric cancer”.

Plasma levels of FAPα were detected by double-antibody sandwich ELISA, the specificity of the capture antibody and the detection antibody were validated by western blot analysis using recombinant human FAPα protein (Supplementary Figure 1A–1B). Figure 1A shows the plasma levels of FAPα in the healthy controls (*n* = 40) and the patients with diverse cancers (*n* = 212) respectively, which included liver cancer (*n* = 37), gastric cancer (*n* = 32), ESCC (*n* = 37), CRC (*n* = 38), nasopharyngeal cancer (*n* = 34), and lung cancer patients (*n* = 34) by ELISA. The mean plasma FAPα values from the ESCC patients (57.55 ± 23.48 ng/ml), CRC patients (52.09 ± 24.63 ng/ml) and liver cancer patients (70.83 ± 30.56 ng/ml) were significantly lower than those from the healthy controls (87.78 ± 44.37 ng/ml) (*P* = 0.008; *P* = 0.004; *P* = 0.033, Figure 1A). While the levels of FAPα in lung cancer patients, gastric cancer patients and nasopharyngeal cancer patients were similar to the healthy control. Extremely low levels of FAPα were found in the ESCC and CRC patient groups.

Furthermore, we expanded the cohort of ESCC patients (*n* = 151) and the benign ESCC (*n* = 36). As shown in Figure 1B, the FAPα levels of patients with benign esophageal disease were higher than those of patients with ESCC (*P* = 0.040) but similar to the healthy controls. The levels of FAPα in early-stage patients (stage I) were lower than those in healthy controls and higher than those in middle and late stage ESCC patients (stages II, III and IV), but showed no significant difference in either healthy controls or in middle and late stage ESCC (Figure 1B). Compared to the healthy controls, significant decline of FAPα levels were present from stage II to stages III and IV (*P* < 0.001 and *P* = 0.008; Figure 1B).

To further assess the levels of plasma FAPα before and after tumor resection, we collected plasma both pre- and post- operation (*n* = 20) and there was no significant difference between preoperative and postoperative FAPα levels.

### Diagnostic value of FAPα in ESCC patients

**Figure 2:**
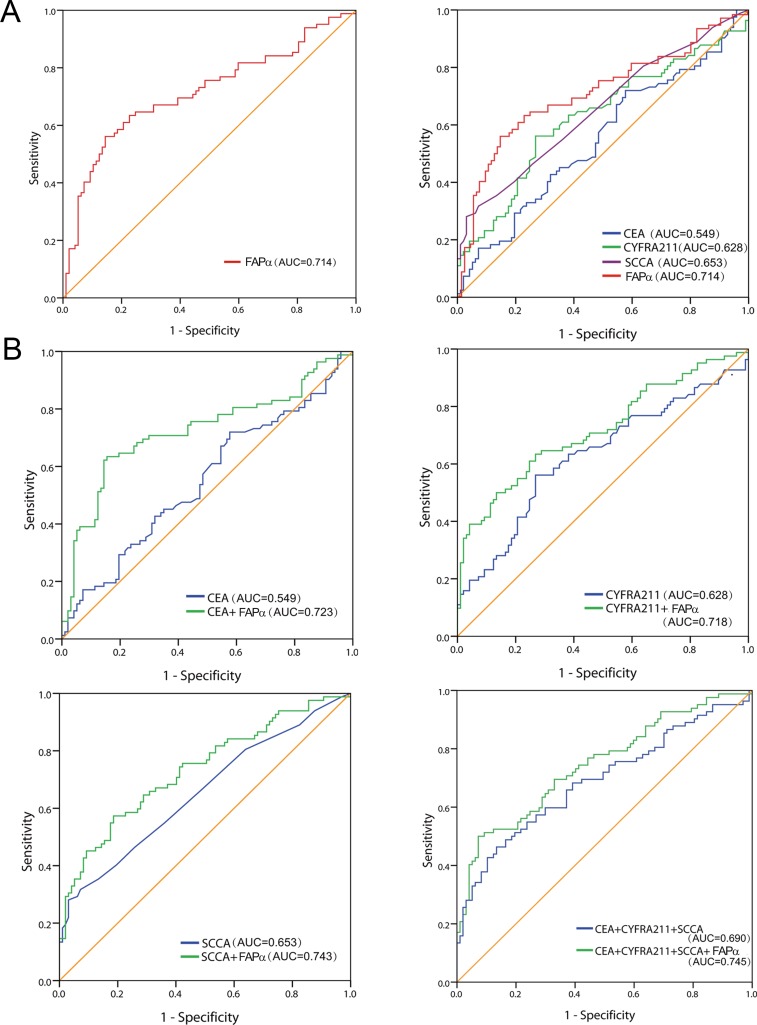
Diagnostic value of plasma FAPα in ESCC patients (**A**) The ROC curves demonstrated the diagnostic strength of FAPα, CEA, CYFR211, SCCA. (**B**) The combined ROC curves of plasma FAPα, CEA, CYFR211, SCCA respectively and the combination of four of them in diagnosis of ESCC against former controls.

The plasma FAPα level was able to distinguish ESCC patients (*n* = 151) from healthy controls (*n* = 194) and the benign ESCC (*n* = 36) with an AUC based on a ROC analysis (AUC = 0.714), whereas the AUCs of the traditional biomarkers of CEA, CYFRA211 and SCCA were 0.549, 0.628, and 0.653, respectively (Figure 2A). The combination of FAPα, CEA, CYFRA211 and SCCA resulted in an improvement in AUC (0.745) compared to the combination of the three traditional biomarkers (AUC = 0.690) (Figure 2B).

However, as shown in Table 1, the sensitivity of FAPα was as high as 56.1%, and the specificity reached 85.6% based on the optimal cut-off, and the sensitivity was 35.4% with the higher adjustment cutoff score (38.6 ng/ml) without compromising the specificity (95.0%); *Moreover*, by combining with CEA, CYFRA211 and SCCA, the sensitivity and specificity reached 50.1% and 92.8% based on the optimal cut-off (0.548), and exhibited a higher sensitivity than combining the three traditional biomarkers alone (32.9%). The findings validated that the effectiveness of plasma FAPα in the diagnosis of ESCC was higher than those of the traditional markers of CEA, CYFRA211 and SCCA.

**Table 1:** Diagnostic values of seprase and traditional biomarker

combinations	Cut off	Sensitivity (%)	Specificity (%)
seprase	49.035	56.1	85.6
	38.575	35.4	95.0
CEA	1.895	72.0	42.3
	5.29	12.2	95.0
CYFRA211	3.035	56.1	73.2
	4.930	19.5	95.0
SCCA	1.600	28.0	96.9
	1.45	29.3	94.0
CEA + seprase	0.516	63.4	84.5
	0.578	37.8	95.0
CYFRA211+ seprase	0.461	63.4	73.2
	0.588	39.0	95.0
SCCA + seprase	0.478	57.3	81.4
	0.603	35.4	95.0
CEA+CYFRA211 +SCCA	0.478	46.3	86.6
	0.594	32.9	95.0
CEA+CYFRA211+SCCA+ seprase	0.548	50.1	92.8
	0.610	41.5	95.0

### Association between plasma FAPα levels of ESCC and HDL-C

The associations between the plasma FAPα levels and the clinicopathological parameters are presented in Table 2. The plasma FAPα levels were not associated with body mass index (BMI) and clinicopathological parameters including age, gender, T classification, lymph node metastasis, and clinical stage.

**Table 2:** Clinical characteristics and seprase levels of the ESCC patients

characteristics	Case numbers	seprase (ng/ml)	
		Median(range)	*P* Value
**BMI**			
< 18.5	28	50.88 (12.43–117.75)	0.860
18.5–25	103	58.95 (13.85–187.43)	
> 25	20	63.50 (27.69–106.22)	
**Age, years**			0.343
< = 62	77	54.36 (12.43–141.57)	
> 62	74	61.47 (13.85–187.43)	
**Gender**			0.152
Male	114	54.79 (12.43–187.43)	
Female	37	67.24 (29.58–141.57)	
**pT status**			0.054
pT1	33	67.75 (17.70–141.57)	
pT2	32	48.45 (24.44–99.15)	
pT3	77	53.76 (12.43–187.43)	
pT4	9	88.16 (41.87–129.57)	
**pN status**			0.880
pN0	79	57.29 (12.43–141.57)	
pN1-3	72	58.42 (13.85–187.43)	
**pTNM status**			0.346
Stage I	29	66.46 (17.70–141.75)	
Stage II	59	51.80 (12.43–129.57)	
Stage III & IV	63	59.44 (13.85–187.43)	

**Figure 3:**
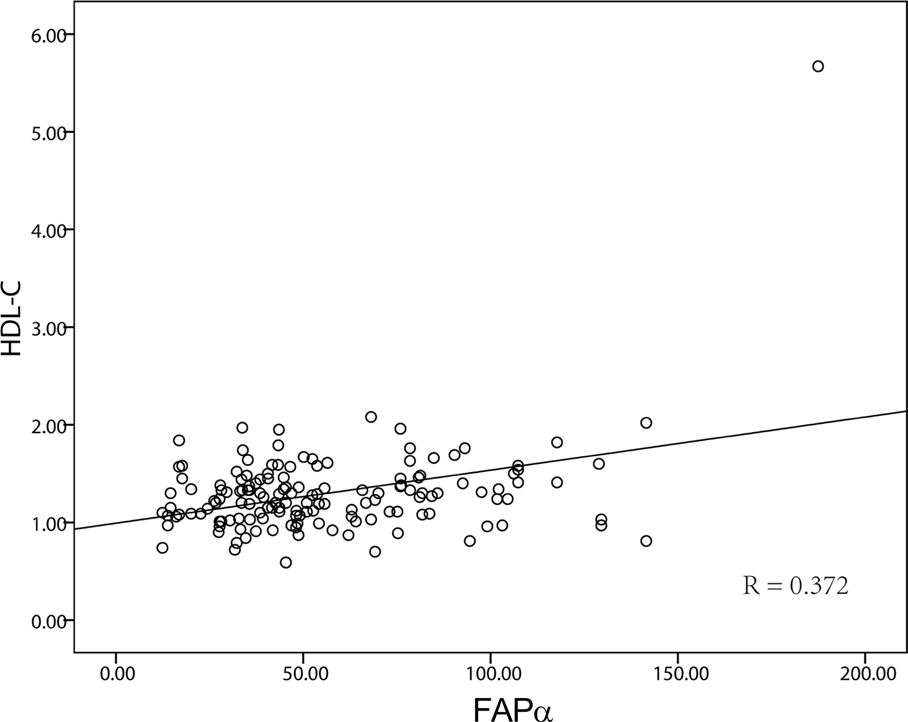
The correlation of FAPα and HDL The expression level of FAPα and HDL-C were significantly positively correlated (*P* < 0.001, *R* = 0.372).

The associations of the plasma FAPα levels with the lipid profile and inflammatory biomarker C-reactive protein (CRP) were presented in Table 3. The plasma FAPα levels were not associated with TG, LDL-C, GLU, apoA1, apoB or CRP, but significantly associated with the HDL-C (*P* < 0.001). Moreover, a partial correlation analysis showed the positive correlation between plasma FAPα levels and HDL-C levels (*R* = 0.372, *P* < 0.001, Figure 3).

**Table 3:** Expression of seprase with different characteristics of biochemical parameters

characteristics		Case numbers	seprase (ng/ml)	
			Median(range)	*P* Value
CRP	0–8.2	120	60.29 (13.85–141.57)	0.198
	> 8.2	31	48.40 (12.43–187.43)	
CHO	1.2–6.47	139	56.40 (12.43–187.43)	0.175
	> 6.47	12	75.87 (28.17–117.75)	
TG	0.2–1.7	127	56.34 (12.43–187.43)	0.360
	> 1.7	24	65.72 (27.88–141.57)	
HDL	< 0.78	7	39.69 (12.43–69.17)	< 0.001
	0.78–2.20	143	57.09 (13.85–141.57)	
	> 2.20	1	187.43	
LDL	< 2.2	15	45.33 (12.43–73.05)	0.222
	2.2–3.4	85	55.07 (13.85–141.57)	
	> 3.4	51	65.93 (16.86–187.43)	
APOA1	< 1.05	31	58.66 (12.43–141.57)	0.991
	1.05–1.76	116	57.67 (13.85–187.43)	
	> 1.76	4	55.82 (43.51–68.12)	
APOB	< 0.63	4	40.28 (12.43–68.12)	0.309
	0.63–1.14	118	55.72 (13.85–141.57)	
	> 1.14	29	68.43 (16.86–187.43)	

The associations of the plasma FAPα levels with the coagulation indices were presented in Supplementary Table 1. The plasma FAPα levels were not associated with PT, PT%, INR, APTT, FBG, TT, DD, FDP. These results indicated that coagulant activity and inflammation may have little effect on the plasma FAPα levels, but lipid profile level is correlated with plasma FAPα in the ESCC patients.

### Characterization of FAPα expression in ESCC tissues

**Figure 4:**
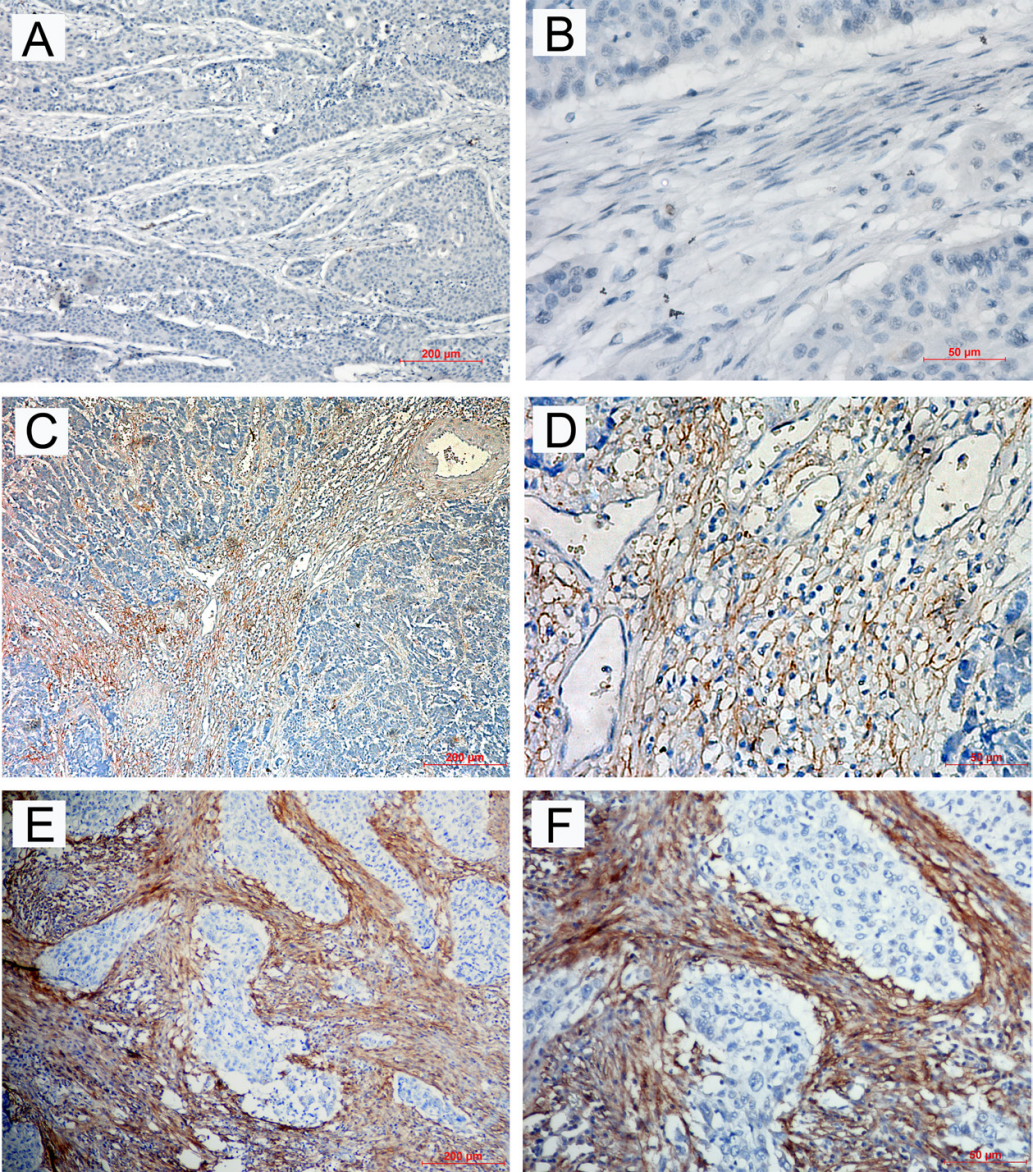
Immunohistochemical staining for FAPα in ESCC tissues The negative expression level (**A**) × 100; (**B**) × 400), low expression level (**C**) × 100; (**D**) × 400) and high expression level (**E**) × 100; (**F**) × 400) of FAPα in ESCC patients.

To evaluate FAPα expression in tumor tissues, we conducted immunohistochemistry with an antibody against human FAPα using prepared paraffin sections from pathological archives. The specificity of FAPα antibody was validated by western blot analysis (Supplementary Figure 1C). FAPα immunoreactivity was observed at varied levels in the stroma of ESCC tissues, but no FAPα immunostaining presented in epithelium of tumour tissues. The major localization was observed in the cytomembrane and/or cytoplasm of the surrounding stromal cells (Figure 4). FAPα was detected in 25 of the 27 ESCC samples (94.6%), and strong expression was detected at 14 samples (54.3%). This result indicated that FAPα was widely expressed in the stoma of ESCC tissues.

### FAPα was expressed and secreted in activated fibroblasts

**Figure 5:**
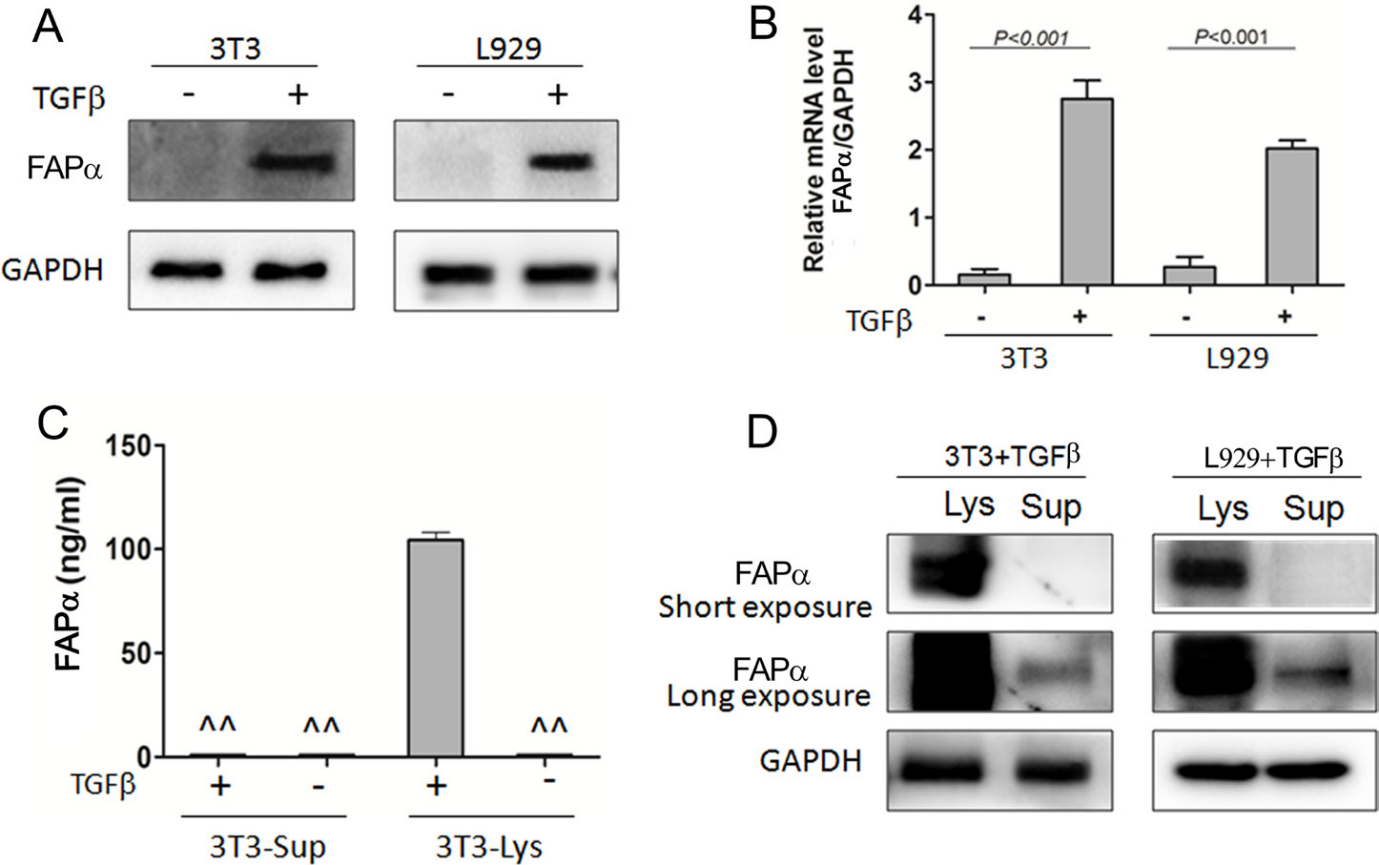
Soluble FAPα detected in the cell supernatant and lysate of fibroblast cell lines (**A**, **B**) FAPα protein and mRNA expression detected by western blot and qPCR in the fibroblast cells, 3T3 and L929. TGFβ (20 ng/ml) was incubated with fibroblast cells for 48 h. (**C**) FAPα e expression detected by ELISA. “^^” means the expression is below the detection limit. (**D**) FAPα expression detected by Immunoprecipitation in the fibroblast cells.

Studies have shown that transforming growth factor-beta (TGFβ) is a potent inducer of fibroblast activation in a variety of cancers [19–21]. In order to identify whether FAPα can express and secrete in activated fibroblasts, qRT-PCR and Western blotting were performed in fibroblast cell lines, 3T3 and L929 with the treatment of TGFβ1. FAPα mRNA and protein were hardly detected in 3T3 and L929 cell lines, but increased significantly in both 3T3 and L929 cells after incubated with TGFβ for 48 h (Figure 5A–5B), whiles 3T3 and L929 cell lines treated with culture supernatant form Eca-109, or co-cultured with Eca-109 cells did not express FAPα. This result exposed that FAPα was expressed in activated fibroblasts but not expressed in normal fibroblasts or tumor cells.

To clarify why FAPα was strongly expressed in CAFs in most of ESCC tissues but was distinctly low in ESCC patients’ blood, ELISA and immunoprecipitation were used to test if FAPα can secrete into the culture supernatant. Soluble FAPα was unable to detect directly in supernatant, no matter TGFβ was added or not (Figure 5C). In contrast, high FAPα expression was detected in the cell lysate when incubated with TGFβ. Furthermore, 8 mL cells supernatant of 3T3 and L929 cells which treated by TGFβ was collected and *lyophilized*. Then, FAPα was detected in the concentrated supernatant by immunoprecipitation. As shown in Figure 5D, FAPα was detected in highly concentrated supernatant of both 3T3 and L929 cells by a Long exposure time of Western blot analysis. These results confirmed that FAPα was expressed with extremely low concentrations in the cell supernatant of activated fibroblasts, and suggested that activated fibroblasts have limited capacity to secrete soluble FAPα.

### The soluble FAPα originated from tumor and other normal tissues

**Figure 6:**
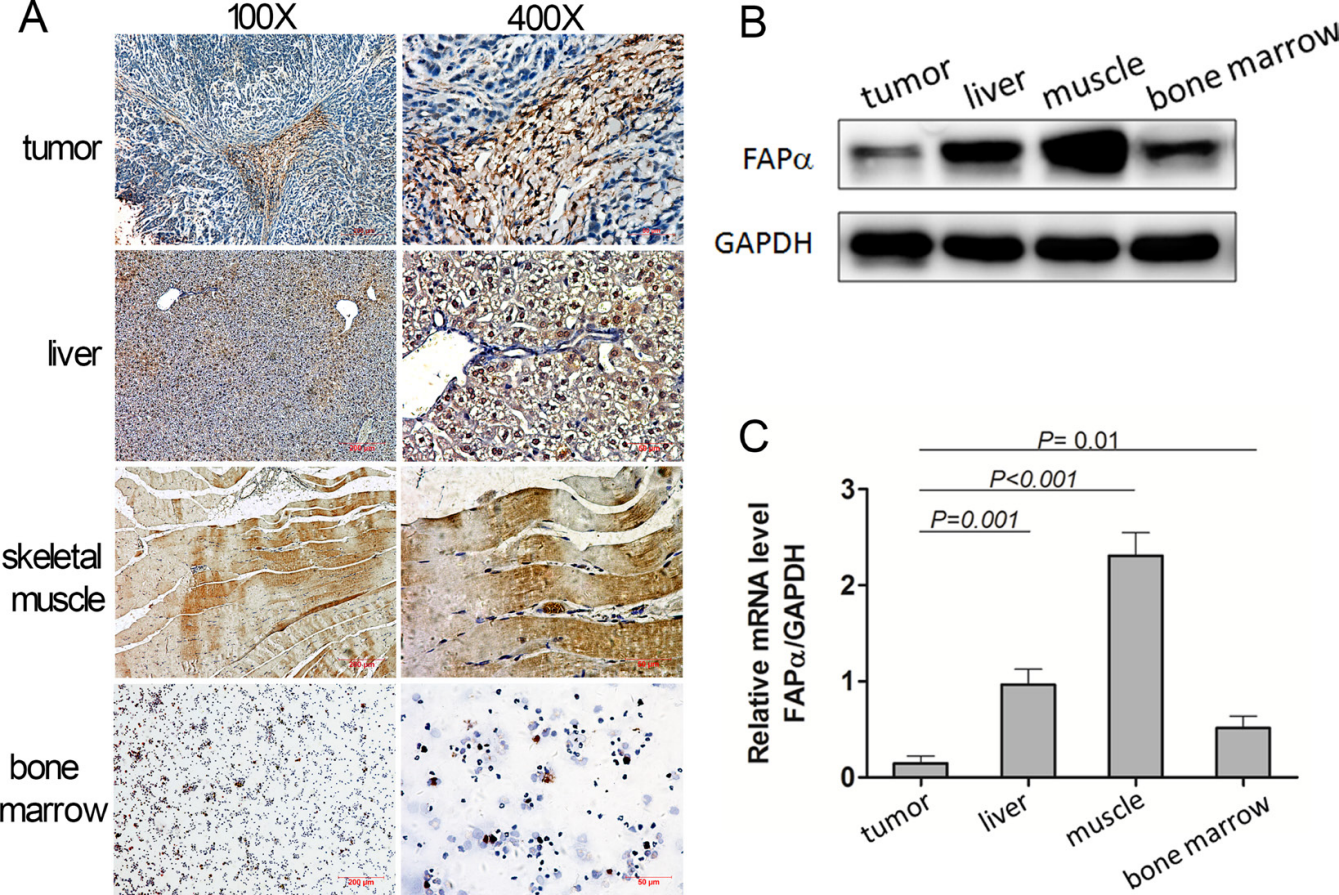
Expression of FAPα detected in the mouse tumor, normal tissues and mouse plasma (**A**) Immunohistochemical staining for FAPα in mouse tumor and normal tissues. (**B**, **C**) FAPα protein and mRNA expression detected by western blot and qPCR in the mouse tumor, mouse liver and mouse muscle.

Next, we wanted to find out where the plasma FAPα came from. We collected plasma both pre- and post- injection of xenograft (*n* = 6) and there was no significant difference between the FAPα levels, although Eca-109 xenograft tissues showed the strong positive expression of FAPα in stromal cell (Figure 6A).

As shown in Figure 6, the FAPα expression was very low in tumor because only the CAFs expressed FAPα but tumor cells didn’t when the tumor consisted of most the tumor cells (Figure 6A). In addition, compared to the tumor tissues, expression of FAPα in bone marrow, liver and skeletal muscle tissues was significantly higher, which disclosed that FAPα were expressed in multiple normal tissues other than only in tumors, and FAPα ^+^ stromal cells in multiple normal tissues might be the physiologic source of the FAPα. Furthermore, Western blotting and qRT-PCR were performed in the Eca-109 xenograft tumor, liver and skeletal muscle tissue homogenate.

## DISCUSSION

In this study, the plasma FAPα levels were found to be significantly reduced in the ESCC patients compared to healthy controls. This was a contradictory conclusion according to extensive evidence showing that high expression of FAPα in tumor tissue [22–26]. Consistent with the finding of this study, multiple reports have showed that plasma FAPα levels were lower in cancer patients compared with healthy volunteers [4, 8, 27], and decline level of circulating FAPα even presented in pancreatic ductal adenocarcinoma which drives formation of the fibroblast-rich desmoplastic stroma [22].

FAPα, as a member of the dipeptidyl prolyl peptidase (DPP) family, has been reported to be a competent diagnostic and therapeutic target because of its high expression in solid tumors and its potential function in tumor invasion [28–31]. In this study, we confirmed that FAPα was overexpressed in human ESCC tissues; This result detected in tumor tissues was contrary to the result detected in the plasma of ESCC patients. In addition, we compared the plasma FAPα level before and after tumor resection and found not significant difference between them. Furthermore, low mouse plasma FAPα levels show inconsistent results with the high expression of FAPα in xenograft tumors tissues, and plasma FAPα levels did not increase along with the tumor growth in our study, which was in line with the result that plasma FAPα had no correlation with the clinicopathological parameters of ESCC patients. Those findings indicated that the FAPα from tumor is not the major contributor to the circulating FAPα.

*FAP*α showed *immunosuppressive* functions in a tumor microenvironment, and depletion of FAPα in stromal cells suppressed the growth of solid malignant tumors [32]. FAPα was reported to be expressed on activated fibroblasts specifically, and could be induced by the important immunosuppressive factor: TGFβ [33]. We examined the expression of FAPα in fibroblasts, and identified that FAPα was induced by the TGFβ. Moreover, FAPα is an integral membrane protein and lacks a secretory signal peptide [34]. Consistently, our study demonstrated soluble form of FAPα in the supernatant of activated fibroblasts was very limited by immunoprecipitation assay. In addition, FAPα was shed from cells as soluble forms *in vitro*, and showed further activation of its proteolytic activity through metalloprotease-mediated truncation [35]. We further examined the expression of FAPα of fibroblasts treated with the supernatant of Eca-109 tumor cells, but no FAPα expression was detected in fibroblasts. Those results confirmed that the FAPα expressed in tumor hardly secreted into the interstitial space or secrete into the plasma, and further showed the circulating FAPα was independent of the tumor.

Although soluble FAPα has also been identified in blood [36–37], the origin of circulating FAPα has not been elucidated. Circulating FAPα is previously speculated to derive from two distinct sources in cancer patients: yet-to-be-identified physiologic source(s), and the tumor [8]. Recently, there is evidence suggesting that the hepatobiliary system and immune cells are the primary physiologic sources of circulating FAPα. In our study, FAPα was present in a wide range of normal tissues, including liver, skeletal muscle and bone marrow in the human xenograft mouse model. Our data is consistent with the study in which plasma soluble FAPα (sFAPα) levels were increased in patients with liver cirrhosis [38]. Though the expression level of FAPα was a little low in normal tissues such as bone marrow, the multi-organ contributed to the high circulating levels of FAPα in healthy individuals. Furthermore, the expression in the muscles was significantly higher than the others. This result exposed that compared to the FAPα expressed in liver and muscle, the FAPα expressed in tumor was negligible. Liver and muscle, especially the muscle, might be the physiologic source of the FAPα. Our results indicated the circulating FAPα was more influenced by the physiologic source than the FAP which expressed in tumor.

Interestingly, the APCE, which circulates in blood and appears structurally similar to FAPα, was identified as a truncated, soluble form of FAPα [36]. Moreover, fibrinolysis inhibitor α2-antiplasmin (α2AP) has been described as a potential *in vivo* substrate of sFAPα. In addition, soluble FAPα levels were reported to reduce in the coronary heart disease (CHD) patient population, but only in the first months after the event, indicating that sFAPα levels may normalize over time [39]. These results indicated that coagulant activity might be compensated for the change of FAPα levels in blood. In our study, however, the plasma FAPα levels were not associated with hemostasis indices. Was it because sFAPα levels change over time? The reason remains to be further explored.

In cancer, lipid and cholesterol homeostasis are often dysregulated to facilitate the cancer cells’ increased demand for these building blocks which are required for proliferation and evasion of apoptosis. Interestingly, our data showed that plasma FAPα was positive correlated (R = 0.372) with HDL-C level in the ESCC patients, but showed no correlation with apoA-I (the major protein component of HDL). Recently, HDL-C levels have reported to be significantly lower in ESCC patients than in normal controls [40]. Low HDL-C level was found to be a risky and prognostic factor of multiple cancers in several epidemiologic studies [41]. Hence, similar with the HDL-C, the lower level of plasma FAPα in cancer patients could possibly be a phenomenon comparable to decreased plasma level of acute-phase proteins discovered in inflammatory processes and malignancy (e.g., low plasma levels of transferrin, albumin, and inter-alpha-trypsin inhibitors) [8], or related to other speculated processes, such as the organ function decline and muscle mass consumption, which indicated the plasma FAPα was a negative acute-phase protein in response to tumor.

Currently, CEA, SCCA and CYFRA211 are the three most commonly used diagnosis markers for ESCC. However, serum tests of the three markers have poor sensitivity. In our study, FAPα showed good sensitivity (56.1%) and specificity (85.6%) in diagnosis of ESCC, and exhibited much higher sensitivity (35.4%) than CEA (12.2%), SCCA (29.3%) and CYFRA211 (19.5%) without compromising specificity (95.0%). Moreover, FAPα combined with the three traditional biomarkers improved the sensitivity (41.5%) without compromising specificity (95.0%) for ESCC detection. Our data were consistent with those studies in which FAPα improved the diagnostic value of CRC [4–5]. These results showd the screen value of FAPα for ESCC, and demonstrated that the combination of FAPα and the three traditional biomarkers can detect about 40% ESCC.

In summary, FAPα is expressed not only in tumors tissues, but also in multiple normal tissues, and FAPα ^+^ cells have very limited ability to secrete soluble FAPα. So circulating FAPα source may come from multiple normal tissues, and lower levels of plasma FAPα in ESCC may be a systemic reaction to the presence of tumor. Furthermore, we found out plasma FAPα can screen ESCC and improve the diagnosis of ESCC by combined with other traditional biomarkers, indicating that circulating FAPα was a potential indicator for the diagnosis of ESCC.

## MATERIALS AND METHODS

### Patients, blood and tissue samples

Plasma was obtained from 326 patients at Sun Yat-Sen University Cancer Center from July 2015 to June 2016, which consisted of liver cancer (*n* = 37, gastric cancer (*n* = 32), ESCC (n = 151), CRC (*n* = 38), nasopharyngeal cancer (*n* = 34), and lung cancer patients (*n* = 34). All cancers were diagnosed based upon the histopathology examination.

The cohort of ESCC patients was consisted of 114 male patients and 37 female patients. The patients ranged in age from 45 to 79 years (mean, 62 years); none had received radiotherapy or chemotherapy prior to surgery. Patients with inflammatory diseases were excluded. 20 paired plasma samples were collected from ESCC patients before tumor resection and 5–7 days post-resection. All plasmas from patients with tumors were collected at the Cancer Center of Sun Yat-sen University at the time of diagnosis and prior to tumor radiation therapy or surgery. The 151 patient characteristics are described in Table 1.

Plasma from 36 patients with benign esophageal disease (21 cases of reflux esophagitis, 6 cases of acute suppurative esophagitis and 9 cases of esophageal hiatal hernia), and plasma from 194 healthy volunteers (144 males, 50 females) without inflammation (ages 36–77 years, mean = 59 years) were collected from Guangzhou First Municipal People’s Hospital from July 2015 to June 2016. Healthy controls samples were matched as closely as possible to the ESCC group with respect to previous handling and the time period of sample collection.

Paraffin-embedded tumour tissue samples were obtained from 34 ESCC patients underwent surgery between May of 2000 and December of 2002. None of the patients had received anticancer treatment prior to surgery, and all of the patients had been histologically confirmed primary ESCC in this retrospective study. The pTNM classification was applied according to guidelines from the 2009 UICC/AJCC Tumor-Node-Metastasis (TNM) classification system. [42].

All plasma samples were stored at -80°C and were measured in 3 months. Each clotted sample was centrifuged at 1,500 g for 10 min. The study was approved by the Ethics Committee of Sun Yat-sen University Cancer Center and Ethics Committee of Guangzhou First Municipal People’s Hospital, informed consent was obtained from each patient.

### Cell lines

The human ESCC cell lines Eca-109, mouse fibroblast cell line NIH 3T3 and L929 (Chinese Academy of Sciences, Shanghai, China) were grown in RPMI 1640 (Invitrogen, USA) supplemented with 10% fetal bovine serum. NIH/3T3 and L929 fibroblasts cells were characterized by the expression of myofibroblasts markers: α-smooth muscle actin (α-SMA) with TGF-β1 treatment (Supplementary Figure 2).

### Xenograft tumor tissues

The six- to eight-week-old BALB/c-nude mice were provided by Guangdong Medical Laboratory Animal Centre (Guangdong, China) and housed under specific pathogen free conditions in the Laboratory Animal Center of Sun Yat-sen University. This study was approved by the ethics committee of Sun Yat-Sen University. The mice were inoculated subcutaneously under the right shoulder with 2 × 10^6^ Eca109 cells. Plasma samples were collected prior to first inoculation and every week after inoculation. After growing for 5 weeks, the animals were sacrificed, and the xenograft tumors, livers and the muscles were removed for use.

### ELISA assay of FAPα

Human plasma FAPα levels were determined by double-antibody sandwich ELISA according to the manufacturer’s instructions (Human FAP DuoSet ELISA, DY3715, R&D systems, USA). Briefly, 96-well microplates were coated with 100 μl/well of the capture antibody (mouse anti-human FAPα antibody, 1 μg/ml, MAB3715) overnight at room temperature and washed thrice with PBS containing 0.05% Tween-20. After blocking with 1% BSA for 1 h at room temperature and another washing step, 100 μl of the test samples (1:50 diluted in 1% BSA) was added and incubated for 2 h at room temperature. Subsequently, after the washing step, 100 μl/well of the detection antibody (biotinylated sheep anti-human FAPα antibody, 200 ng/ml, BAF3715) was added and incubated for 2 h at room temperature. After the washing step, 100 μl/well of Streptavidin-HRP (1:200) was added and incubated for 20 min at room temperature. Washing thrice again and finally, the substrate (tetramethylbenzidine) solution was added 100 μl/well, and the reaction was terminated using 2 N H_2_SO_4_ and read at an OD of 450 nm. S32 SsSSSsSamples and standards were measured in duplicate. In each experiment, a seven point standard curve was generated using 2-fold serial dilutions of rhFAP from 4,000 pg/mL to 62.5 pg/mL in reagent diluent and a four parameter logistic curve-fit for each ELISA plate was constructed to calculate corresponding FAPα concentrations in individual samples. Each test included a standard control (CV = 12%). The operator performing the ELISA was blinded regarding the patient characteristics of samples analyzed.

Mouse plasma and soluble FAPα of mouse fibroblast cell line were determined by double-antibody sandwich ELISA using Mouse FAP ELISA kit (CUSABIO, China, CSB-EL008424MO) according to the manufacturer’s instructions.

### Tumor markers, biochemical and hemostasis indices assay

The concentrations of CEA and CYFRA211 in the plasma were assessed using electrochemiluminescence immunoassay (ECLIA) kits (Roche, Germany) on a Roche E170 fully automatic electrochemistry luminescence immunity analyzer (Roche, German). The levels of SCCA in the plasma were detected using an ARCHITECT I2000SR immune analyze system (Abbott, America). Each test included a standard control (CV < 5%).

The plasma biochemical indices including TG, CHO, HDL-C, LDL-C(WAKO, Japan), ApoA1, Apo-B(Maccura,China) and inflammatory biomarker CRP (WAKO, Japan) were measured on an automatic biochemical analyzer (LABOSPECT 008; Hitachi HighTechnologies Corporation, Japan).

The plasma hemostasis indices including PT, APTT, FBG, TT, DD (Siemens, Germany) and FDP (BIOLINKS CO., LTD, Japan) were measured on an automatic coagulation analyzer (SYSMEX CS-5100 Hemostasis System, Japan). PT% and INR were calculated according to the formula recommended by the manufacture’s instruction.

### RNA extraction and real-time quantitative PCR

Total RNA was extracted from cell lines using the Trizol reagent (Invitrogen, USA) according to the manufacture’s instruction. Reverse transcription of total RNA (800 ng) was done using SuperScript II reverse transcriptase. The quantification of target and reference gene (GAPDH) were performed in triplicate on a LightCycler^®^ 480 II (Roche, Applied Science) using a SYBR green-based assay (BioRad, USA). Expression data were normalized to the geometric mean by housekeeping gene GAPDH as an internal control. The primers used in the real-time RT-PCR reaction were as follows: FAPα, 5′-ATCTATGACCTTAGCAATGGAGAATTTGT-3′ (forward), 5′-GTTTTGATAGACATATGCTAATTTACT CCCAAC-3′ (reverse); and GAPDH, 5′-GACTCATGACC ACAGTCCATGC-3′ (forward) and 5′-AGAGGCAGGGA TGATGTTCTG-3′ (reverse).

### Immunoprecipitation (IP)

After the lyophilization of cells supernatant, PBS was used to dissolve the powder. Then FAPα antibody (1:40, AF3715, R&D, USA) was added in the PBS buffer and shaked at 4°C overnight. Protein A+G Agarose (Beyotime Biotechnology, China) was added for 4 h and then centrifuged at 1000 g, 5 min for 5 times. Next, equivalent protein amounts were denatured in an SDS sample buffer and were ready for the Western blot analysis.

### Western blot analysis

Total protein was extracted using a lysis buffer and protease inhibitor (Beyotime Biotechnology, China). Equivalent protein amounts were denatured in an SDS sample buffer, and then were separated by SDS-PAGE and transferred onto polyvinylidene difluoride mem-brane. After being blocked with 5% non-fat dry milk in PBS containing 0.05% Tween-20, the blotted membranes were incubated with anti-human FAPα antibody (1:2000, AF3715, R&D, USA) and secondary antibody (1:5000, Boster, China) thereafter. GAPDH protein levels were also determined by using the specific antibody (1:1000, Boster, China) as a loading control.

### Immunohistochemistry

The paraffin-embedded tissues were sectioned into 4-μm-thick sections. The sections were dewaxed, rehydrated and rinsed. The antigens were retrieved by heating the tissue sections at 100°C for 20 min in citrate (10 mmol/L, pH 6.0) solution when necessary. The sections were subsequently immersed in a 3% hydrogen peroxide solution for 10 min to block endogenous peroxidase activity and were incubated with the primary antibody sheep anti-human FAP (1:200, AF3715, R&D, USA) at 4°C overnight. A negative control was performed by replacing the primary antibody with PBS. The sections were then incubated with a horseradish peroxidase labeled secondary antibody (1:100, Boster, China) at room temperature for 120 min. Finally, the signal was developed for visualization with 3, 3′-diaminobenzidine tetrahydrochloride, and all of the slides were counterstained with hematoxylin.

### Statistical analysis

All statistical analyses were carried out using the SPSS 16.0 statistical software package (SPSS Inc., Chicago, IL, USA). The Mann-Whitney U test was used to evaluate the difference in plasma FAPα, concentrations between tumor patients and healthy controls. Pearson’s chi-squared test was used to analyze the relationship between FAPα levels and patients’ clinical parameters’ characteristics. The efficacy of diagnosis for ESCC was evaluated by the area under receiver operating characteristic (ROC) curve (AUC). The cut-off values were defined as the value either with the maximization of the Yuden index or the 90% specificity. Furthermore, sensitivity, specificity were used to compare the efficiency of diagnosis. All statistical tests were two-side, *P* < 0.05 was considered to be statistically significant in all cases.

## SUPPLEMENTARY MATERIALS FIGURES AND TABLES


