# Supplementary material for: Evaluation of the circulating level of fibroblast activation protein α for diagnosis of esophageal squamous cell carcinoma
Source: Oncotarget. 2017 Mar 16;8(18):30050–62. doi: 10.18632/oncotarget.16274 (PMC5444725; doi:10.18632/oncotarget.16274)
Supplement: Supplementary file 1 [file oncotarget-08-30050-s001.pdf]

Evaluation of the circulating level of fibroblast activation protein α for diagnosis of esophageal squamous cell carcinoma

Supplementary Materials

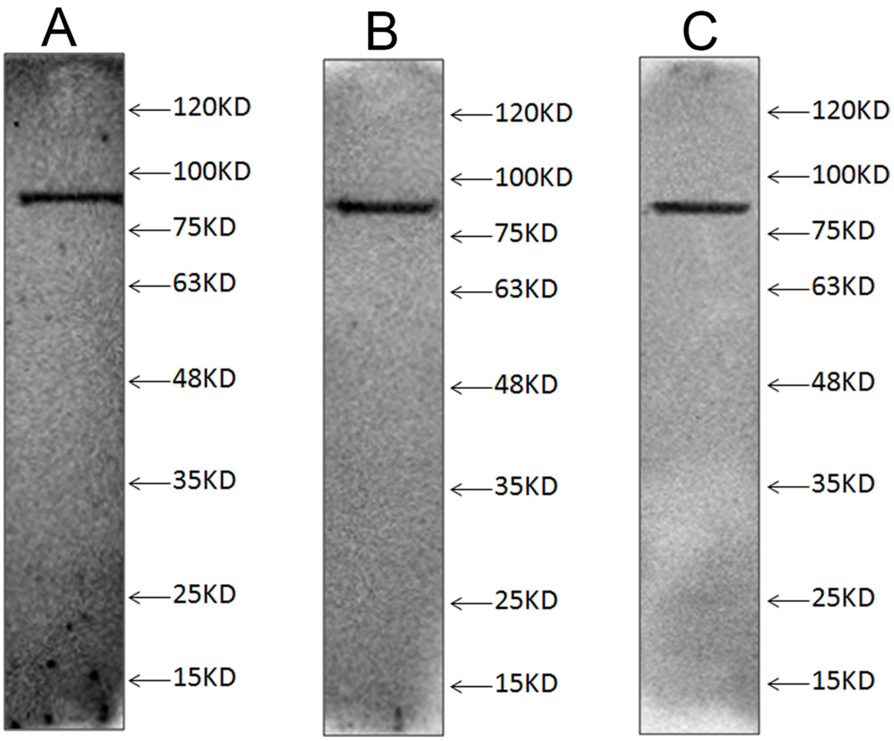

Supplementary Figure 1: Validation of antibody specificity. (**A**) FAPα ELISA capture antibody (**B**) FAPα ELISA detection antibody (**C**) FAPα antibody was tested by western blot for using recombinant human FAPα protein.

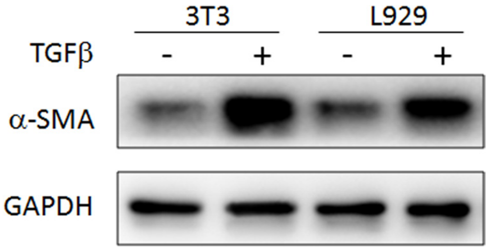

Supplementary Figure 2: Validation of characteristic of cell lines. α-SMA expression was detected by western blot in the fibroblast cells, 3T3 and L929. TGFβ (20 ng/ml) was incubated with fibroblast cells for 48 h.

Supplementary Table 1: The associations of the plasma seprase levels with the coagulation indices

characteristics		Case numbers	FAPa (ng/ml)	
			Median (range)	*P* Value
PT	< 10.5	32	58.35 (14.59–107.36)	0.882
	10.5–13.5	119	56.97 (12.43–187.43)	
PT%	< 70.0	1	34.93	0.793
	70.0–130.0	135	57.75 (12.43–187.43)	
	> 130.0	15	55.74 (31.75–102.15)	
INR	< 0.85	1	75.94	0.833
	0.85–1.20	149	57.14 (12.43–187.43)	
	> 1.20	1	48.79	
APTT	< 22.5	22	58.61 (27.88–107.36)	0.981
	22.5–34.0	125	57.12 (12.43–187.43)	
	> 34.0	4	53.99 (34.93–73.05)	
FBG	< 1.80	6	44.91 (31.75–54.19)	0.783
	1.80–4.00	108	57.12 (12.43–129.57)	
	> 4.00	37	59.55 (14.59–187.43)	
TT	14.0–21.0	147	57.53 (12.43–187.43)	0.666
	> 21.0	4	47.08 (38.48–55.68)	
DD	0.00–0.55	101	56.82 (12.43–187.43)	0.560
	> 0.55	50	61.92 (20.09–117.75)	
FDP	0.0–5.0	128	56.01 (12.43–187.43)	0.149
	> 5.0	23	72.45 (16.86–117.75)	
